# Circadian Regulation of Immunity Through Epigenetic Mechanisms

**DOI:** 10.3389/fcimb.2020.00096

**Published:** 2020-03-13

**Authors:** Ricardo Orozco-Solis, Lorena Aguilar-Arnal

**Affiliations:** ^1^Laboratorio de Cronobiología y Metabolismo, Instituto Nacional de Medicina Genómica, Mexico City, Mexico; ^2^Departamento de Biología Celular y Fisiología, Instituto de Investigaciones Biomédicas, Universidad Nacional Autónoma de México, Mexico City, Mexico

**Keywords:** circadian rhythm, chromatin, epigenetics, transcriptional regulation, infection

## Abstract

The circadian clock orchestrates daily rhythms in many physiological, behavioral and molecular processes, providing means to anticipate, and adapt to environmental changes. A specific role of the circadian clock is to coordinate functions of the immune system both at steady-state and in response to infectious threats. Hence, time-of-day dependent variables are found in the physiology of immune cells, host-parasite interactions, inflammatory processes, or adaptive immune responses. Interestingly, the molecular clock coordinates transcriptional-translational feedback loops which orchestrate daily oscillations in expression of many genes involved in cellular functions. This clock function is assisted by tightly controlled transitions in the chromatin fiber involving epigenetic mechanisms which determine how a when transcriptional oscillations occur. Immune cells are no exception, as they also present a functional clock dictating transcriptional rhythms. Hereby, the molecular clock and the chromatin regulators controlling rhythmicity represent a unique scaffold mediating the crosstalk between the circadian and the immune systems. Certain epigenetic regulators are shared between both systems and uncovering them and characterizing their dynamics can provide clues to design effective chronotherapeutic strategies for modulation of the immune system.

## Introduction

The immune response is perhaps the most evident organismal response against infection, and mounting research depicts the circadian system as a critical regulator of immune defense. Circadian rhythms are endogenous processes with an oscillatory pattern following a daily cycle, mostly controlled by the circadian system. Circadian clocks are hierarchically organized, with the central pacemaker located in the suprachiasmatic nucleus (SCN) in the brain, and subordinate clocks in almost all peripheral tissues (Hastings et al., 2018). Through sensing and integrating light signaling, the SCN adjust its time to external day/night cycles and in turn provides cues to synchronize peripheral clocks. At the cellular level, a molecular clock machinery is expressed and sustains circadian oscillations in cellular functions such as gene expression, protein translation, intracellular signaling, metabolism, and many cell type-specific functions (Takahashi, 2017). At this regard, the molecular clock is present in distinct types of immune cells, and the circadian and immune systems interact in many manners to set the time of multiple processes controlling the immunological surveillance and response to infection (Man et al., 2016; Westwood et al., 2019). Indeed, nearly every aspect of the immunity, both innate and adaptive, display a daily oscillatory pattern, including immune cell trafficking and circulating humoral components, inflammatory processes, responses to infection, expression of cytokines, chemokines and recognition receptors, signaling, amongst others (Scheiermann et al., 2013; Man et al., 2016). However, the molecular gears implicated in the collaborative interactions between the circadian and immune systems remain largely uncovered. Interestingly, fundamental mechanisms contributing to rhythmicity occur in the chromatin fiber, through oscillations in the genome functions including transcription (Takahashi, 2017; Pacheco-Bernal et al., 2019). Hundreds of genes are daily transcribed, most of them regulating critical processes to sustain homeostasis. The rhythmic transcriptome is assisted by a specific epigenetic machinery that cooperates with the molecular clock to impose transcriptional oscillations (Orozco-Solis and Sassone-Corsi, 2014; Pacheco-Bernal et al., 2019). Intriguingly, these circadian gene regulatory mechanisms have been shown to contribute to a broad number of processes within the immune system. Hence, in this minireview we postulate that epigenetic mechanisms are at the crossroad between the circadian and the immune systems. We first provide some insights on the epigenetic regulation of circadian rhythms. Then, we dissect known examples reinforcing this idea at four fundamental levels: physiology of the immune cells, host-pathogens interactions, the inflammatory response and the adaptive immune system.

## Epigenetic Mechanisms Underlying Circadian Rhythms

**Figure 1:**
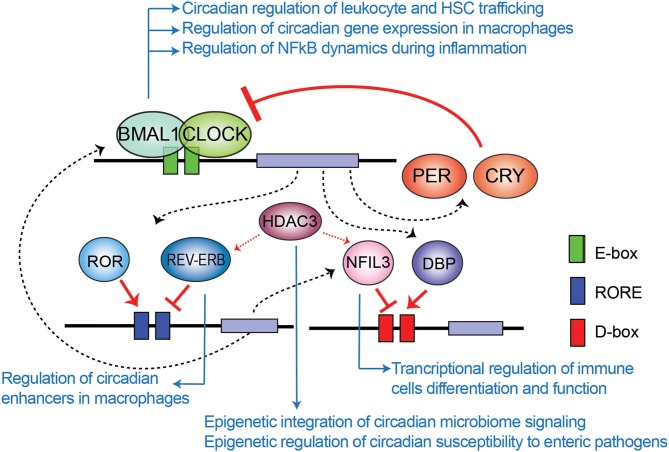
Transcriptional-translational feedback loops control circadian gene expression. Rhythmic binding to e-boxes on chromatin of the clock components of the positive loop, CLOCK:BMAL1, induce the expression of clock-controlled genes and the clock negative regulators PER and CRY. Additionally, the nuclear receptor REV-ERBα and ROR impose transcriptional rhythms on genes via RORE cis regulatory elements, while the PAR-bZIP transcription factors DBP, and the repressor NFIL3 interplay to drive transcriptional rhythms in a set of genes through binding to D-boxes. Blue arrows relate molecular components of the clock TTFL involved in epigenetic regulation of the indicated mechanisms of immunity and infection, which are further discussed across the text.

The mammalian circadian clock machinery is present in nearly all cells in the organisms and consist of interconnected and autoregulated feedback loops driving cyclic transcription of a set of genes, named clock-controlled genes (CCGs). The core components of this machinery are the clock proteins, mostly transcriptional regulators. Within the positive loop, the heterodimer CLOCK:BMAL1 rhythmically binds to e-boxes at regulatory regions in the genome, hereby inducing architectural changes in chromatin states to promote transcription of CCGs and the clock repressors *Period* (*Per1*-*3*) and *Cryptochrome Cry1*-*2* genes (Figure 1). PER and CRY proteins conform a repressor complex which opposes CLOCK:BMAL1 driven transcriptional activation, hence silencing CCGs. Additional loops interlock, as described for the nuclear receptors Rev-Erbα and ROR, which bind to ROR-elements at *Bmal1* promoter to drive rhythmic transcription of the gene. Also, some CCGs are transcription factors (TFs) themselves, such as the PAR (proline and acidic amino acid–rich) basic leucine zipper (bZIP) TF family including DBP (D-box binding protein), TEF (thyrotroph embryonic factor) and HLF (hepatic leukemia factor), which impose circadian transcription to subordinate genes. Remarkably, the coordinated action between all these transcriptional regulators orchestrates a tissue or cell type-specific circadian transcriptome, and constitutes the molecular basis of circadian rhythmicity (Takahashi, 2017). Notably, post-transcriptional mechanisms and post-translational modifications of clock proteins regulate circadian function, and confer means to attend intracellular signaling, as evidenced for example by adjustment of the oscillatory period length by phosphorylation of PER and CRY proteins mediated by the casein kinase family [extensively reviewed elsewhere (Kojima et al., 2011; Hirano et al., 2016; Wong and O'Neill, 2018)].

Transcriptional oscillations within the chromatin fiber are tightly regulated by a particularly dynamic epigenome (Pacheco-Bernal et al., 2019). Indeed, chromatin conformation and function has been the subject of intense research for decades, and the relevance of epigenetic control in determining cellular fate has long been recognized (Allis and Jenuwein, 2016; Baldi et al., 2020). Epigenetic approaches significantly advance our understanding of gene regulation in health and disease, and we can now explain functionality for many epigenomic features. Epigenetic mechanisms include histone post-translational modifications, DNA methylation events, chromatin conformation and transitions defining accessibility or specific non-coding RNAs, and excellent reviews can be found in Allis et al. (2015), Allis and Jenuwein (2016), Berdasco and Esteller (2019), and Klemm et al. (2019). Because the molecular clock drives daily transcriptional oscillations, a tight cooperation with the regulatory epigenome is necessary. For example, in the mouse liver the transcriptional activating histone post-translational modifications (PTMs) H3K4me3 and H3K9ac appear rhythmic at promoters of CCGs, and their abundance is antiphasic to the repressive H3K9me3 and H3K27me3 marks (Koike et al., 2012; Takahashi, 2017). The coordinated action between the molecular clock and epigenetic remodelers appear to underlie circadian chromatin transitions (Pacheco-Bernal et al., 2019). For example, rhythmic histone acetylation is achieved by recruitment of CLOCK:BMAL1 together with p300 and CBP histone acetiltransferases (HATs) (Etchegaray et al., 2003; Lee et al., 2010), and PER complexes can recruit the H3K9 methyltransferase SuVar3-9 to assist repression (Duong and Weitz, 2014). Also, spatial configuration of the genome and rhythmic interactions between CCG promoters and their distal regulatory elements are essential mechanisms to sustain transcriptional oscillations, and both BMAL1 and REV-ERBα regulate the 3D chromatin conformation during the circadian cycle (Aguilar-Arnal et al., 2013; Kim et al., 2018). Remarkably, metabolic cues can be integrated by specific sensors which in turn translate this information to the chromatin fiber in a circadian manner, as is the case for the nicotinamide adenine dinucleotide (NAD^+^)- dependent histone deacetylase (HDAC) SIRT1 (Sirtuin1) (Berger and Sassone-Corsi, 2016). At this regard, SIRT1 HDAC activity is circadian due to rhythmic biosynthesis of its cofactor NAD^+^, hence acetylation levels of SIRT1 targets oscillate (Nakahata et al., 2009; Ramsey et al., 2009). For example, the H3K4 methyltransferase MLL1 is a SIRT1 target, and rhythmic deacetylation controls its activity in a way that deposition of the H3K4me3 epigenetic mark oscillates at CCG promoters such as *Dbp, Per2*, or *Cry1* (Aguilar-Arnal et al., 2015). In summary, epigenetic mechanisms significantly contribute to define the clock transcriptional output in a tissue-specific manner. This orchestrates daily fluctuations in the chromatin conformation and compaction, but also constitutes a platform to rapidly respond and adapt to environmental challenges such as infection. In the next sections we discuss how the epigenetic component of the circadian clock contributes to the immune function.

## Clock-Controlled Physiology of Immune Cells via Epigenetic Mechanisms

### Epigenetic Regulation of Circadian Trafficking of Immune Cells

**Figure 2:**
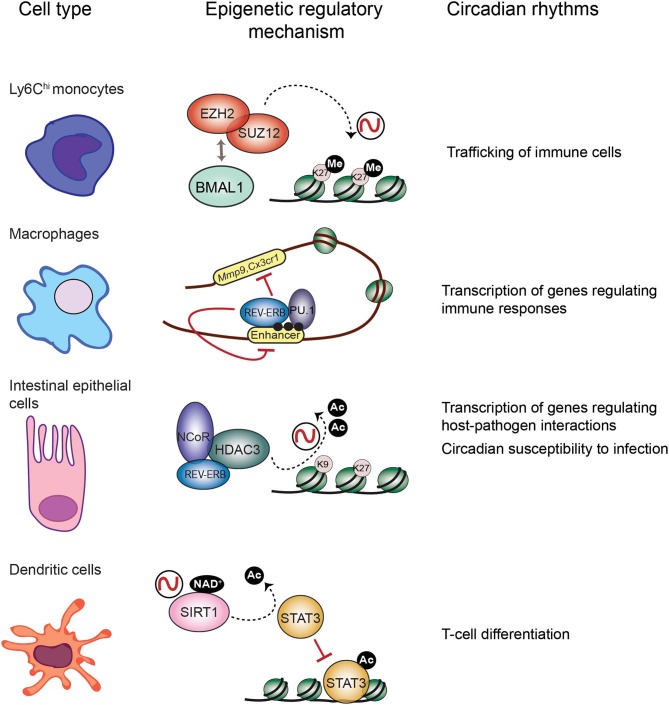
Epigenetic regulatory mechanisms at the core of circadian function in the immune system. Circadian trafficking in the bloodstream is observed for many types of immune cells. In Ly6Chi inflammatory monocytes, circadian trafficking is regulated by an epigenetic mechanism involving molecular interactions between the circadian TF BMAL1 and the Polycomb repressive complex to regulate levels of the H3K27 methylation at the promoters of chemokine genes. In macrophages, transcription of regulatory genes appears cyclic, and the clock component REV-ERBα could be responsible by inhibiting enhancer function together with the TF PU.1, and their location is coincident with the enhancer mark H3K4me1 (black dots). Intestinal epithelial cells are components of the mucosal barrier in the gut, hence they participate in host-pathogens interactions. In these cells, the repressor complex REB-ERBα-NCoR-HDAC3 rhythmically deacetylate histones to promote chromatin compaction and cyclic silencing of genes involved in host pathogens interactions, and this mechanism could also participate in regulating circadian responses to infection. Differentiation of dendritic cells is orchestrated by the TF STAT3. The circadian HDAC SIRT1 deacetylates and inactivates STAT3, and this mechanism could be implicated in circadian regulation of differentiation and proliferation of T cells.

It has been extensively described that the immune response is tightly controlled by the circadian clock (Scheiermann et al., 2013). Persistent oscillations in immune cell trafficking in the blood evidences the intervention of circadian rhythms in immunity. For example, the number of circulating neutrophils, monocytes and lymphocytes in the peripheral blood fluctuate around the day in humans and mice (Haus and Smolensky, 1999; Dimitrov et al., 2009; Scheiermann et al., 2012). Interestingly, circadian-controlled neural signals influence leukocyte recruitment to tissues under homeostasis or inflammatory conditions (Scheiermann et al., 2012). Indeed, rhythmic recruitment of hematopoietic cells to skeletal muscle and bone marrow can be entrained by photic cues and are completely abolished in *Bmal1*^−/−^ mice, underscoring the importance of the molecular clock in leukocyte trafficking (Scheiermann et al., 2012). Also, mouse inflammatory monocytes Ly6C^hi^ trafficking into tissues varies in a diurnal manner with a ~3-fold difference, while patrolling monocytes Ly6C^low^ do not (Nguyen et al., 2013). Remarkably, diurnal rhythms of Ly6C^hi^ depend on BMAL1, and are necessary for a proficient immune response to infection (Nguyen et al., 2013). Along this line, myeloid cell specific deletion of *Bmal1* gene eliminates the Ly6C^hi^ monocyte rhythm, rendering mice prone to death by sepsis (Nguyen et al., 2013; Man et al., 2016). Interestingly, this deletion also promotes expression of the chemokine genes *Ccl2, Ccl8*, and *S100a8*, because BMAL1 recruits members of the Polycomb repressive complex (PRC) to their promoters, such as the H3K27 methyltransferases EZH2 and SUZ12 and their partner EED (Nguyen et al., 2013; Aranda et al., 2015) (Figure 2). Hence, H3K27me3 mark appears rhythmic at these genes' promoters, while in the absence of BMAL1, RNA Pol II, and H3K4me3 are favored, driving their transcriptional oscillations (Nguyen et al., 2013). Along these lines, EZH2 is an critical epigenetic regulator of hematopoiesis and B-cell differentiation, and regulates clock function in cultured cells through epigenetic mechanisms (Etchegaray et al., 2006; Herviou et al., 2016), suggesting that the molecular interplay between the clock machinery and PRC to regulate cyclic transcription might further extend to other immune cells.

Haematopoietic stem cells (HSCs) and their progenitors also circulate in the bloodstream in physiological conditions, showing marked circadian fluctuations in their trafficking (>2-fold), both in mice and humans (Lucas et al., 2008; Méndez-Ferrer et al., 2008). These fluctuations are abolished in *Bmal1*^−/−^ mice, relay on circadian sympathetic innervation activity, and photic cues entrain them (Lucas et al., 2008; Méndez-Ferrer et al., 2008; Weger et al., 2017). Intriguingly, haematopoietic stem and progenitor cells (HSPC) release from the bone marrow (BM) during the early resting period coincides with a reduction in production of the chemokine CXCL12 by the BM mesenchymal stromal cells. CXCL12 and its receptor, CXCR4, are critical for colonization, homing and maintenance of HSPC in the BM, thereby daily fluctuations in CXCL12-CXCR4 axis signaling trigger HSPC circadian mobilization (Méndez-Ferrer et al., 2008; Nagasawa, 2014). Remarkably, CXCL12 expression and function is tightly regulated by the O_2_ sensor HIF1α TF, in a way that discrete hypoxic regions within the BM show increased CXCL12 expression and HSC cell tropism (Ceradini et al., 2004; Morikawa and Takubo, 2016). Moreover, deletion of HIF1α in mouse HSCs impairs their natural quiescence state and maintenance upon transplantation (Takubo et al., 2010). Indeed, fluctuations in O_2_ levels are effective synchronizers of the molecular clock machinery, and a large number of genes related to rhythmic processes are directly regulated not only by BMAL1 recruitment to their promoters, but also by co-occupancy by HIF1α in hypoxic conditions, indicating that clock and hypoxia signaling present reciprocal regulation at the chromatin level (Adamovich et al., 2017; Wu et al., 2017). While the epigenetic mechanisms coordinating the interplay between BMAL1 and HIF1α remain largely obscure, recent research points toward JMJC (Jumonji-C) family lysine demethylases KDM5A, KDM6A, and KDM3A as effective oxygen sensors (Batie et al., 2019; Chakraborty et al., 2019; Qian et al., 2019). Along these lines, early hypoxia increases H3K4me3 epigenetic mark in human fibroblasts due to inactivation of KDM5A, and many genes with upregulated H3K4me3 are related to hypoxia signaling (Batie et al., 2019). KDM5A may participate in repression of cytokines such as CXCL12, thus allowing its expression in hypoxic conditions. Interestingly, KDM5A has been shown to interact with CLOCK:BMAL1 heterodimers in human cells to regulate *Per2* transcription (DiTacchio et al., 2011). Also, KDM6A is essential for proper HSPC migration and normal hematopoiesis, while KDM3A promotes fitness within the mesenchymal stromal cells in the BM (Thieme et al., 2013; Huang et al., 2019). Taken together, these evidences support the idea that hypoxia signaling and circadian rhythms might coordinate dynamic changes in the epigenome to generate transcriptional responses controlling HSC physiology and functionality.

Indeed, 24-h rhythms in immune cell trafficking have further consequences both in disease and treatment. For example, myeloid cell recruitment to atherosclerotic lesions oscillates, presenting a peak at the onset of the resting phase, while myeloid cell adhesion to microvascular beds happens with opposite phase (Winter et al., 2018). Hence, time-of-day specific inhibition of leukocyte recruitment provides means to treat atherosclerotic lesions without affecting inflammatory processes in the microcirculation (Winter et al., 2018). In this scenario, it would be interesting to determine which epigenetic components of the circadian clock are involved in mediating time of day-specific immune cell trafficking and recruitment, as they could provide new therapeutic targets for diseases involving the immune system, and foundation to design chronotherapeutic strategies.

### The Circadian Clock in Macrophages Control Enhancer Activity and the Transcriptional Program

The molecular clock sustains rhythmic expression of hundreds of genes in every tissue, implicating rhythmic fluctuations in the epigenome. Growing research define the role of epigenetic remodelers in regulating circadian function in many tissues or cell types. However, the way the circadian clock and the epigenome converge to regulate the immune functions is just emerging. At the cellular level, eight percent of all transcripts in mouse peritoneal CD11b^+^ macrophages are expressed with a circadian rhythm (Keller et al., 2009), many of which are implicated in response to infection. Amongst these, members of the stress response such as *Hspa1, Hspd1*, or *Hspa5*, genes implicated in immune regulation including *Cd59a, Cd69, Cd86, Cd200r1*, and *4*, components involved in phagocytosis such as *Vamp8* or scavenger receptors, and certain adhesion molecules oscillate in a circadian fashion (Keller et al., 2009). Interestingly, when cultured mouse bone marrow-derived macrophages (BMDMs) are activated through the TLR4 receptor, *Bmal1* deletion profoundly alters the NF-κB signaling pathway, and extends the inflammatory response trough overexpression of proinflammatory genes (Oishi et al., 2017). Moreover, in these TLR4 activated macrophages, BMAL1 protein binds to genomic sites coincident with the presence of the TFs PU.1 and NF-κB (Oishi et al., 2017). Interestingly, PU.1 appears central to shape the genomic landscape in macrophages and B cells, by initiating nucleosome remodeling and directing the enhancer mark H3K4me1 deposition, thus determining functional enhancers through collaborative interactions with additional TFs (Ghisletti et al., 2010; Heinz et al., 2010). In LPS-induced TRL4 activation, some of these TFs are the stress inducible NF-κB and IRF family members (Ghisletti et al., 2010). Recruitment of the HAT p300 occurs at active enhancers, including those activated by LPS (Visel et al., 2009; Ghisletti et al., 2010), although the mechanisms enabling this recruitment remain mostly unknown. Interestingly BMAL1's C-terminal transactivation domain activates rhythmic transcription by interacting with the KIX domain of p300, hereby recruiting the HAT to specific sites in the genome and promoting histone H3 K9/K14 acetylation (Etchegaray et al., 2003; Garg et al., 2019). Yet, loss of BMAL1 in macrophages results in increased H3K27ac epigenetic mark, associated with active enhancers, underscoring that a mechanism involving a repressor instead of an activator is compromised (Rada-Iglesias et al., 2011; Oishi et al., 2017).

Mounting research points to the negative regulator of circadian transcription Rev-Erbα, as a major repressor of enhancer activity in several cell types, including macrophages (Lam et al., 2013; Kim et al., 2018) (Figure 2). In BMDMs, Rev-erbα probably opposes transcription of enhancer RNAs (eRNAs), a subset of small non-coding RNAs which are hallmarks of active enhancers (Wu and Shen, 2019). Similarly to BMAL1, in mouse BMDMs Rev-Erbα is mostly bound at enhancer elements, coincident with the H3K4me1 epigenetic mark and the PU.1 pioneer TF (Lam et al., 2013) (Figure 2). Specific deletion of Rev-erbα/β in macrophages results in increased expression of some genes including *Mmp9* and *Cx3cr1*, and a significant increase of eRNA expression and H3K9ac mark at their distal regulatory elements (Lam et al., 2013). Additionally, Rev-Erbα is a direct repressor of several genes controlling innate immunity in macrophages, such as *Il6, Il19, Cxcl6, Cxcl11*, and *Ccl2* (Gibbs et al., 2012; Sato et al., 2014). Along the same lines, BMAL1 is a direct positive regulator of *Nrf2* gene transcription in mouse BMDMs, and NRF2 is a master TF inducing the expression of antioxidant genes such as *Hmox1, Gsr*, or *Nqo1* (Early et al., 2018). Thereby, rhythmic expression of NRF2 and suppression of oxidative stress could be related to rhythmic inflammatory mechanisms observed for example in production of IL-1β (Early et al., 2018).

## Host-Pathogens Interactions

Host-pathogen interactions conform a highly dynamic system in which each part rapidly evolves; pathogens intend to defeat the host defenses and hijack the cellular components needed for infection while the host phenotypically adjusts and responds to counter such threats. In this scenario, epigenetic mechanisms emerge as fast and highly plastic gears which could define the outcome of host-pathogen interactions (Gómez-Díaz et al., 2012). Also, the circadian system incorporates a very significant epigenetic component to the clockwork to drive transcriptional oscillations; however, we still know little about the extent and significance of the rhythmic epigenome in host-pathogen interactions. Remarkably, circadian rhythms dictate the outcomes of infection. For example, mice differentially respond to *Salmonella enterica* infection at daytime or nighttime, showing striking differences in inflammatory response and cecum gene expression depending on the time-of-day when infection initiates (Bellet et al., 2013). Similarly, viral infections are enhanced in *Bmal1*^−/−^ mice (Edgar et al., 2016), while the magnitude of infection by distinct protozoan parasites, including *Plasmodium* and *Leishmania major* also presents significant variation along the day (Kiessling et al., 2017), or even in response to time of feeding (Hirako et al., 2018; Prior et al., 2018). Interestingly, *Leishmania* secretes a histone H3 mimic, LmaH3, in infected cells which is incorporated into human chromatin, providing evidence that the genome structure and function in the host is a direct target of the pathogens' mechanisms of infection (Dacher et al., 2019). Whether *Leishmania* histone hijacking of the host's chromatin depends on the time of the day remains to be elucidated.

### NFIL3 and HDAC3 Relay Inputs From Intestinal Microbes to the Host Genome

The TF NFIL3, also known as E4BP4, is emerging as a link between the circadian clock and the host's susceptibility to intestinal pathogens. NFIL3 is a basic leucine zipper TF which represses transcription through binding to D-boxes in the genome, hence counteracting the function of the PAR-bZIP TF family such as DBP (Figure 1). These TFs are clock-controlled, and their molecular interplay drive circadian transcription of a set of genes (Mitsui et al., 2001; Ueda et al., 2005). Interestingly, *Nfil3*^−/−^ mice are susceptible to infection by the enteric pathogens *Citrobacter rodentium* and *Clostridium difficile*, probably due to a defect in the production of Type 3 innate lymphoid cells (ILC3) or T helper 17 cells (T_h_17), which participate in defense mechanisms on mucous membranes (Yu et al., 2013; Geiger et al., 2014). Remarkably, *Nfil3* transcription oscillates in intestinal epithelial cells, and these rhythms are governed by the core clock repressor Rev-Erbα, and by the intestinal microbiome, as their amplitude is dampened in germ free mice (Wang et al., 2017). Hence, bacterial signals at barrier surfaces are integrated through multiple regulatory layers convergent in NFIL3 and circadian function. Notably, epithelial NFIL3 controls expression of a circadian lipid metabolic program which is also regulated by the histone deacetylase HDAC3 (Alenghat et al., 2013; Wang et al., 2017; Dávalos-Salas et al., 2019). Along these lines, intestinal HDAC3 expression is induced by gut microbes and is rhythmically recruited to chromatin together with the corepressor NCoR and the clock protein Rev-Erbα, directly imposing diurnal rhythms in H3K9ac and H3K27ac at the regulatory regions of genes involved in lipid metabolism and nutrient transport (Kuang et al., 2019) (Figure 2). Hence, HDAC3 emerges as a key epigenetic effector that integrates inputs from intestinal microbes and circadian rhythms and relays them to host metabolic genes (Kuang et al., 2019). Interestingly, intestinal epithelial cell expression of HDAC3 also confers protection to *Citrobacter rodentium* infection (Navabi et al., 2017). Further research will be needed to understand the molecular interplay between HDAC3 and NFIL3 to control circadian intestinal homeostasis through signal integration from the gut microbes. Finally, it will be interesting to determine the epigenetic role of HDAC3-NCoR-Rev-Erbα complex in the control of circadian susceptibility of the host to enteric pathogens.

## The Inflammatory Response Triggered by NF-κB

### The Molecular Clock and NF-κB Cooperate in the Chromatin Fiber

The nuclear factor binding near the κ light-chain gene in B cells, or NF-κB, is a master transcriptional regulator of hundreds of genes implicated in inflammatory responses (Zhang et al., 2017). In non-pathological conditions, NF-κB is generally sequestered to the cytoplasm through interaction with a set of inhibitors pertaining to the “Inhibitor of κB” (IκB) family (Zhang et al., 2017). Activation of NF-κB can be triggered by infectious threats, and results in rapid translocation into the nucleus and binding to regulatory elements for inflammatory target genes, including Il-1 and Ccl2/Ccl7/Ccl11 gene clusters (Barish et al., 2010). Yet, at each tissue or cell type, the inflammatory response triggered by NF-κB is unique, and specific for the stimulus due to the cooperative chromatin binding of NF-κB with other TFs (Smale et al., 2014). Remarkably, mounting evidence relates the circadian positive regulators of transcription, CLOCK:BMAL1, to NF-κB-dependent inflammatory responses. For example, in the mouse liver, a set of enhancers bound by CLOCK:BMAL1 remain inactive in normal conditions, and these are highly enriched in NF-κB binding motif (Trott and Menet, 2018). Notably, CLOCK and the p65 subunit of NF-κB, and BMAL1 and the RelB subunit of NF-κB physically interact *in vitro* and in cell lines (Bellet et al., 2012; Spengler et al., 2012). Remarkably, after LPS stimulation, hepatic p65 relocates to the promoters of clock repressor genes such as *Per1, Per2*, and *Cry2*, and the H3K27ac activator epigenetic mark decreases at these sites, hereby repressing them (Hong et al., 2018). Moreover, acute inflammation induces a genome wide re-localization of CLOCK:BMAL1 to sites in proximity to genes involved in the immune system response or interferon signaling, most of them bound by p65 and with increased H3K27ac and polymerase II (Hong et al., 2018). Consistently, chronic inflammatory responses such as those associated with unbalanced diets are accompanied by a re-localization of p65 to core clock repressor genes, including *Per1* and *Rev-erb*α, which in turn decrease their transcription, while CLOCK:BMAL1 complexes become active at sites associated with the immune response in the mouse liver (Hong et al., 2018). Contrary, when mice are subjected to healthy eating patterns such as calorie restriction, the hepatic circadian expression of NF-κB signaling pathways is reinforced, probably contributing to the beneficial effects of calorie restriction (Fabbiano et al., 2016; Sato et al., 2017; Wu et al., 2019). Taken together, these evidences demonstrate a tight interplay between the molecular clock and NF-κB in the chromatin context, to control circadian responses to infection. It will be necessary to dissect the epigenetic regulators contributing to this interplay, and specifically the long-range interactions directing the transcriptional reprogramming associated to inflammatory responses to determine the relevance of circadian enhancers.

## The Adaptive Immune System and the Circadian Clock: An Epigenetic Link

Antigen presentation triggers the adaptive immune response in which different cell types participate. Epigenetic mechanisms on the antigen-presenting cells have a pivotal role on regulating expression of essential effectors. For example, in dendritic cells (DC) histone deacetylation is crucial for differentiation (Nencioni et al., 2007). Also, the TF signal transducer and activator of transcription 3 (STAT3) controls dendritic function by regulating target genes involved in DC differentiation, such as the IDO enzyme and the HLA class II genes (Sun et al., 2009; Cacalano, 2016; Kitamura et al., 2017). Importantly, the circadian HDAC SIRT1 is a master regulator of STAT3 function by deacetylation, hereby inactivating it (Nie et al., 2009) (Figure 2). Interestingly, SIRT1 signaling in DC orchestrates T helper 1 (T_H_1) and regulatory T-cell (Treg) differentiation (Liu et al., 2015), while CD8+ T cells activation depends on inhibition of *Sirt1* expression, which is accompanied by increased histone acetylation at the *Tbx21* (T-bet) locus and a metabolic switch toward oxidative reactions (Kuroda et al., 2011). Hereby, it would be possible that this control is modulated by the circadian clock, as the nicotinamide adenine dinucleotide (NAD^+^)- dependent SIRT1 HDAC activity is circadian by the fluctuating levels of NAD^+^ due to the circadian expression of the enzyme NAMPT, the rate-limiting enzyme on the NAD^+^ biosynthesis (Nakahata et al., 2008). Accordingly, CD8+ and CD4+ T cells show circadian fluctuation in proliferation, which is dampened in *Clock* mutant mice (Fortier et al., 2011; Druzd et al., 2017). Moreover, a circadian transcriptional program is apparent in CD8+ T cells, which allows them to respond more efficiently to antigen presentation during daytime, by rhythmically modulating downstream effectors of the T cell receptor, such as IRF4, AKT, and mTOR pathways (Nobis et al., 2019). At tissue level, lymphocytes including T cells CD8+, CD4+, and B cells show circadian oscillation in the entry/egress from the lymph nodes, and BMAL1 is necessary for this function by directing the circadian expression of key regulators of lymphocyte trafficking such as *Ccr7* and *S1pr1* (Druzd et al., 2017). Interestingly, histone acetylation at *Ccr7, S1pr1*, and *S1pr4* promoters is modulated by HDAC3 in Tregs (Wang et al., 2015), while in B cells, *Hdac3* deletion impairs VDJ recombination, implying epigenetic mechanism of chromatin conformation in the production of fully recombined B-cell receptor (Stengel et al., 2017). Whether the epigenetic remodeler complex HDAC3-NCoR-RevErbα regulates VDJ recombination should be addressed to find out the possibility of a direct link with the clock machinery in this crucial element of the adaptive immunity. Finally, sleep disturbance appears to be related with a reduction in NK cells, and sleep modulates immunological memory in humans (Geiger et al., 2015; Suzuki et al., 2017), while in *Drosophila* the gene *nemuri* is a neuronal antimicrobial peptide that also promotes sleep (Toda et al., 2019). All together, these findings reinforce the notion that the circadian clock is necessary to the induction of appropriate adaptive immune responses.

## Concluding Remarks

Responses to infection require big amounts of energy and resources to adequately fight threats, however these responses need to be balanced to avoid compromising critical organism functions. The circadian clock regulates daily rhythms in immune functions in normal conditions; however, during infection circadian regulation becomes critical to temporally organize the immune response and make it more effective and compatible with the host vital functions. The clock machinery are mostly transcriptional regulators which act in the chromatin fiber, and many epigenetic mechanisms are shared between the circadian and the immune systems. The circadian TFs BMAL1 and RevErbα regulate immune cell trafficking by directing the expression of chemokines involved in mobilization, and to do so they cooperate with chromatin modifiers such as members of the Polycomb family, or regulate active enhancers. Histone acetylation also plays a prominent role in orchestrating a circadian transcriptome in regulatory cell types for the immune response, while inflammatory processes are coordinated by multiple interactions between NF-κB and the clock TFs. At this regard, it will be necessary to further dissect the interactions between metabolism and the epigenome during the circadian cycle in immune cells, as it will provide means to understand how metabolic cues regulate immunity. Finally, it is important to note that much of our understanding in the circadian aspects of immunity has been studied in rodents, thus advancing this knowledge in humans will be essential for opening possibilities of translation and, specifically, to design adequate strategies in circadian medicine (Munch and Kramer, 2019; Ruben et al., 2019).

## Author Contributions

RO-S and LA-A wrote the paper.

### Conflict of Interest

The authors declare that the research was conducted in the absence of any commercial or financial relationships that could be construed as a potential conflict of interest.
